# Therapy-related acute myeloid leukemia following treatment of lymphoid malignancies

**DOI:** 10.18632/oncotarget.13262

**Published:** 2016-11-10

**Authors:** Sarah Bertoli, Arthur Sterin, Suzanne Tavitian, Lucie Oberic, Loïc Ysebaert, Reda Bouabdallah, François Vergez, Audrey Sarry, Emilie Bérard, Françoise Huguet, Guy Laurent, Thomas Prébet, Norbert Vey, Christian Récher

**Affiliations:** ^1^ Service d'Hématologie, Centre Hospitalier Universitaire de Toulouse, Institut Universitaire du Cancer de Toulouse Oncopole, Toulouse, France; ^2^ Université Toulouse III Paul Sabatier, Toulouse, France; ^3^ Cancer Research Center of Toulouse (CRCT), UMR1037 INSERM, ERL5294 CNRS, Toulouse, France; ^4^ Service d’Hématologie, Institut Paoli-Calmettes, Marseille, France; ^5^ Laboratoire d’Hématologie, Centre Hospitalier Universitaire de Toulouse, Institut Universitaire du Cancer de Toulouse Oncopole, Toulouse, France; ^6^ Service d’Epidémiologie, Centre Hospitalier Universitaire de Toulouse, Toulouse, France; ^7^ UMR 1027, INSERM-Université de Toulouse III, Toulouse, France; ^8^ Département d'Oncologie Moléculaire, Centre de Recherche en Cancérologie de Marseille (CRCM), Institut Paoli-Calmettes, UMR1068 Inserm, Marseille, France; ^9^ Aix-Marseille University, Marseille, France

**Keywords:** leukemia, therapy-related acute myeloid leukemia, second cancer, lymphoma, chronic lymphocytic leukemia

## Abstract

Therapy-related acute myeloid leukemia (t-AML) is a heterogeneous entity most frequently related to breast cancer or lymphoproliferative diseases (LD). Population-based studies have reported an increased risk of t-AML after treatment of lymphomas. The aim of this study was to describe the characteristics and outcome of 80 consecutive cases of t-AML following treatment of LD. t-AML accounted for 2.3% of all AML cases, occurred 60 months after LD diagnosis, and were characterized by a high frequency of FAB M6 AML and poor-risk cytogenetic abnormalities. Time to t-AML diagnosis was influenced by patient age, type of LD, and treatment. Among the 48 t-AML patients treated with intensive chemotherapy, median overall survival (OS) was 7.7 months compared to 26.1 months in *de novo*, 4.2 months in post-myeloproliferative neoplasm, 9.4 months in post-myelodysplastic syndrome, 8.6 months in post-chronic myelomonocytic leukemia AML, 13.4 months in t-AML secondary to the treatment of solid cancer, and 14.7 months in breast cancer only. OS of post-LD t-AML patients was significantly influenced by age, performance status, myelodysplastic syndrome prior to LD/t-AML, and treatment regimen for LD. Thus, t-AML following lymphoid malignancies treatment should be considered as very high-risk secondary AML. New treatment strategies in patients with LD/t-AML are needed urgently.

## INTRODUCTION

Therapy-related acute myeloid leukemia (t-AML) is described in the WHO classification [1–2] as AML occurring after a previous cancer whose treatment comprises mainly an alkylating agent, ionizing radiation therapy – giving rise to chromosome 5 or 7 abnormalities, and/or topoisomerase II inhibitor – generating balanced translocations, including t(15;17) [3] or 11q23 [*MLL*] rearrangements among others. The poor prognosis of t-AML was first reported in patients with multiple myeloma treated with melphalan [4]. Subsequently, almost all other alkylating agents have been associated with an increased risk of t-AML in patients treated for a wide spectrum of diseases [5] whereas topoisomerase II inhibitors were first associated with secondary AML in children who received epipodophyllotoxins for acute lymphoblastic leukemia [6]. Individualization of this category of AML permitted to emphasize the distinct biological and clinical features of this entity typically characterized by an adverse outcome [7].

Although most of the knowledge of the relationship between prior exposure to anti-cancer therapy and therapy-related AML comes from epidemiological studies, arguments for a direct impact of cytotoxic therapy in leukemogenesis exist. In therapy-related acute promyelocytic leukemia, mitoxantrone, etoposide, and doxorubicin have been shown to induce DNA cleavage at specific genomic hot spot sites in *PML* and *RARA* genes, leading to t(15;17) translocation. Moreover, mitoxantrone has been associated with secondary AML in patients treated for a nonmalignant condition [8]. More recently, it has been shown that cytotoxic therapy favors the expansion of rare hematopoietic stem cells carrying *TP53* mutations in a founding clone that expands preferentially after therapy and evolves to leukemia through the accumulation of chromosomal abnormalities found typically in t-AML [9].

The most frequent therapy-related myeloid neoplasms are related to breast cancer and lymphoproliferative diseases (LD) [10–12]. Data from large US population-based cancer registries have shown that t-AML is 4.7 fold more frequent than expected in the general population, which is consistent with other nationwide registries [10–14]. These population-based studies reported an increased risk of t-AML after treatment of non-Hodgkin lymphomas (NHL) (Standardized Incidence Ratio, 5.96 for follicular lymphoma; 4.96 for diffuse large B cell lymphoma) [15]. This risk remains elevated for at least one decade after chronic lymphocytic leukemia (CLL), Hodgkin lymphoma (HL), and NHL treatment [10, 16]. It has also been described that the risk of AML has significantly increased in the last 30 years after chemotherapy for NHL, remained stable for HL, and decreased for multiple myeloma, consistent with changing practices and differential leukemogenicity of specific therapies [10]. Alkylating agents and topoisomerase II inhibitors have long-standing constituted the backbone of T or B-cell lymphomas treatments, accounting for a certain proportion of t-AML. More recently, nucleoside analogs – mainly fludarabine - and anti-CD20 monoclonal antibodies – firstly rituximab, among others, have enlarged treatment options and have been used widely, thus improving outcome [17]. Whether this new therapeutic landscape has modified the spectrum of this subset of t-AML remains to be studied in detail.

Since previous neoplasia and treatment are highly heterogeneous in the t-AML category, the aim of this study was to describe the characteristics and outcome of AML following treatment of lymphoid malignancies.

## RESULTS

### Characteristics of LD at diagnosis

In the 1997–2012 period, 80 consecutive cases of t-AML following treatment of NHL, HL, T-cell lymphoma (TCL), and chronic lymphocytic leukemia (CLL) (thereafter referred as lymphoid diseases; LD) were diagnosed. Median age at LD diagnosis was 60 years (IQR [interquartile range], 48–67). Thirteen patients (16%) had diffuse large B cell lymphoma (DLBCL), five had mantle cell lymphoma (MCL) (6%), two had Burkitt-like lymphoma (BL) (3%), fourteen had follicular lymphoma (FL) (18%), two had MALT/SALT lymphoma (3%), one had lymphocytic lymphoma (1%), 11 had HL (14%), eight had CLL (10%), and five had TCL (6%) (Table 1). The majority of them had advanced stages of disease. The median number of prior LD treatment lines was one (1–6); 30 patients (38%) had two treatment lines and nine patients (11%) had three or more treatment lines. Among the 74 patients who received chemotherapy, 11 (15%) received a fludarabine-based regimen (all of them in association with an alkylating agent), 34 (46%) a CHOP-like regimen, 19 (26%) chlorambucil, and 34 (46%) rituximab. Autologous stem-cell transplantation (ASCT) was performed in 16 patients (22%).

**Table 1:** Characteristics of previous LD

Age at LD diagnosis – years (median, IQR)	60 (48–67)
**Center – n (%)**	
Toulouse	35 (44)
Marseille	45 (56)
**LD type – n (%) Aggressive NHL**	**22 (28)**
DLBCL	13 (16)
MCL	5 (6)
BL	2 (3)
Other	2 (3)
**Indolent NHL**	**32 (40)**
FL	14 (18)
Chronic lymphocytic leukemia	8 (10)
MALT/SALT	4 (5)
Lymphocytic	1 (1)
Unknown	1 (1)
**Hodgkin lymphoma**	**11 (14)**
**T-cell lymphoma**	**5 (6)**
**Not known**	**2 (3)**
**Stage at diagnosis – n (%)**	
Localized*	14 (18)
Advanced**	46 (58)
Binet A	1 (1)
Binet B-C	5 (6)
Unknown	14 (18)
**Treatment – n (%)**	80 (100)
**Chemotherapy only**	21 (26)
**Radiotherapy only**	1 (1)
**Chemotherapy + radiotherapy**	19 (24)
**R-chemotherapy**	28 (33)
**R-chemotherapy + radiotherapy**	6 (8)
**Unknown**	5 (6)
Chemotherapy agent***	
Alkylating agents	66/68 (97)
Fludarabine-based	11/68 (16)
CHOP-like	34/68 (50)
Chlorambucil	19/68 (28)
Rituximab	34/70 (49)
**No. of treatment lines (median, min-max)**	1 (1–6)
2 lines - n (%)	30 (38)
≥ 3 lines – n (%)	9 (11)
**Autologous stem cell transplantation – n (%)**	16 (20)

Abbreviations: LD: lymphoid disease; NHL: non-Hodgkin Lymphoma; DLBCL: diffuse large B cell lymphoma; MCL: mantle cell lymphoma; BL: Burkitt-like; FL: follicular lymphoma; NOS: not otherwise specified.

*Localized is defined by Ann-Arbor classification 1–2; ** Advanced is defined by Ann-Arbor stage III-IV; ***At first line or later.

### Characteristics of LD-therapy-related AML

LD-therapy-related AML (LD/t-AML) accounted for 2.3% of all cases of AML treated with intensive chemotherapy in the studied period. Median time from LD diagnosis to t-AML diagnosis was 60 months (IQR, 31–106). Median age at t-AML diagnosis was 66 years (IQR, 57–71). Characteristics of LD/t-AML are described in Table 2. ECOG performance status was ≥ 2 in 20 patients (25%). FAB classification was mainly M2 (36%), M4 (16%), and M6 (11%). There was no diagnosis of APL. Median WBC at diagnosis was 3.8 x 10^9^/L (IQR, 1.7–29.5) and median bone marrow blast was 46% (IQR, 32–69). Cytogenetic risk was favorable for three patients (4%), intermediate for 36 patients (45%), and adverse for 29 patients (36%). *MLL* rearrangements, chromosome 5 and 7 abnormalities, were observed in 6, 16, and 22% of cases respectively, whereas 22% had complex karyotypes and 22% monosomal karyotypes. These poor-risk cytogenetic abnormalities were two-fold more frequent than in *de novo* AML (Table 2). The characteristics at diagnosis of *de novo* AML (*n* = 829), AML secondary to myelodysplastic syndrome, Philadelphia-negative myeloproliferative neoplasm or chronic myelomonocytic leukemia (*n* = 292) and t-AML following treatment of solid cancers (*n* = 85) diagnosed in the 2000–2012 period are presented in Table 2.

**Table 2:** Characteristics of LD/t-AML at diagnosis and comparison to other therapy-related AML, secondary AML and *de novo* AML

	LD/t-AML *n* = 80	Other t-AML *n* = 85	sAML^§^*n* = 292	*De novo* AML *n* = 829
History of previous cytopenia* n (%)	22 (28)#	13 (15)	NA	61 (7)
History of previous documented MDS** – n (%)	17 (21)	NA	235 (81)	–
Delay to secondary AML*** – months (median, IQR)	60 (31–106)	NA	NA	–
**Age at AML diagnosis - years (median, IQR)**	66 (57–71)	67 (58–74)	72 (63–78)	62 (48–72)
**ECOG PS – n (%)**				
0–1	36 (45)	55 (65)	115 (39)	523 (63)
2–3–4	20 (25)	17 (20)	71 (24)	150 (18)
Not known	24 (30)	13 (15)	106 (36)	156 (19)
WBC - .10^9^/L (median, IQR)	3.8 (1.7–29.2)	3.3 (1.7–16.4)	6.2 (2.1–23.1)	7.9 (2.6–38.8)
Platelet count - .10^9^/L (median, IQR)	48 (23–85)	50 (34–98)	62 (30–132)	67 (37–120)
Bone marrow blasts − % (median, IQR)	46 (32–69)	46 (30–76)	35 (24–60)	63 (37–83)
FAB - n (%)				
0	6 (8)	3 (4)	10 (3)	41 (5)
1	4 (5)	13 (15)	21 (7)	180 (22)
2	26 (33)	40 (47)	146 (50)	280 (34)
3^§§^	0	1 (1)	0	13 (2)
4	13 (16)	14 (17)	37 (13)	137 (17)
5	8 (10)	2 (2)	19 (7)	99 (12)
6	9 (11)	6 (7)	15 (5)	21 (3)
7	1 (1)	3 (4)	5 (2)	13 (2)
Not defined	10 (12)	2 (2)	12 (4)	26 (3)
Unknown	3 (4)	1 (1)	27 (9)	19 (2)
**Karyotype – n (%)**				
Favorable	3 (4)	3 (4)	0	74 (9)
Intermediate	36 (45)	40 (47)	157 (54)	541 (65)
Adverse	29 (36)	38 (45)	100 (34)	180 (22)
Unknown	12 (15)	4 (5)	35 (12)	21 (3)
*MLL*+^%^	4 (6)	12 (14)	3 (1)	24 (3)
Del 5 / 5q^%^	11 (16)	15 (18)	49 (17)	79 (10)
Del 7 / 7q^%^	15 (22)	9 (11)	59 (20)	91 (11)
Complex^%^	15 (22)	19 (22)	57 (20)	107 (13)
Monosomal^%^	15 (22)	16 (19)	46 (16)	82 (10)

Abbreviations: MDS: myelodysplastic syndrome; FAB: French-American-British classification; *MLL*: Mixed-lineage leukemia.

§ AML secondary to Philadelphia-negative myeloproliferative neoplasm, myelodysplastic syndrome and chronic myelomonocytic leukemia.

§§ LAM3 were collected only from 2011 in our database, which implies an underrepresentation of this category.

* Defined as anemia, thrombocytopenia, neutropenia or any combination after end of LD treatment and more than six months before AML diagnosis – lymphopenia excluded.

** Defined as documented MDS diagnosed more than six months before AML diagnosis.

*** Defined as (AML diagnosis date – LD diagnosis date).

# For three of them, cytopenia occurred after four to seven cycles of R-chemotherapy.

% These abnormalities were present either alone or combined.

### Impact of LD characteristics and their treatment on LD/t-AML

Age at LD diagnosis had a major impact on time from LD diagnosis to LD/t-AML diagnosis, which was longer for patients younger than 60 years old (86.2 months vs. 40.1 months; *P* = 0.003). Time from LD diagnosis to LD/t-AML diagnosis was shorter for TCL, lymphocytic lymphoma, DLBCL and BL (13.8, 15.6, 23.6 and, 37.4 months respectively), intermediate for FL, MALT/SALT, MCL and CLL (63.5, 65.5, 69.1 and 57 months respectively), and longer for HL (95.6 months). Table 3 this difference in latency could in part be explained by the shorter survival of patients treated for T-NHL or aggressive NHL. Time to LD/t-AML was not significantly different when considering fludarabine-based regimens, chlorambucil, or ASCT (58.4, 62.4, and 93.5 months, respectively). However, this interval was significantly shorter after CHOP-like or R-containing regimens (33.6 months, *P* = 0.02 and 48.6 months, *P* = 0.009, respectively). Neither subtype of LD nor specific regimen used for LD treatment (including rituximab regimen vs. others) influenced the cytogenetic profile of LD/t-AML. However, there was a trend for an increased incidence of monosomal karyotypes in patients who received an ASCT (38% vs. 14%, *P* = 0.07). 53% of patients presented one or more cytopenias between end of treatment of lymphoma and diagnosis of LD/t-AML. For these patients, time to LD/t-AML was shorter (31.7 vs. 79 months; *P* = 0.04). In three cases, cytopenias occurred during lymphoma treatment: one case of neutropenia and two cases of thrombocytopenia occurred after 4, 4 or 7 cycles, or R-chemotherapy, leading to treatment withdrawal or dose reduction. For these three patients, time to AML was even shorter (26.1 months). Seventeen patients (21%) developed a documented myelodysplastic syndrome (MDS) at least six months before t-AML diagnosis.

**Table 3:** Time from LD diagnosis to LD/t-AML diagnosis according to LD type

LD type – n (%)	Median latency (months)
**DLBCL**	23.6
**MCL**	69.1
**BL**	37.4
**Other**	69.8
**FL**	63.5
**CLL**	57
**MALT/SALT**	65.5
**Lymphocytic**	15.6
**Unknown**	87.4
**Hodgkin lymphoma**	95.6
**T-cell lymphoma**	13.8
**Not known**	NA

Abbreviations: DLBCL: diffuse large B cell lymphoma; MCL: mantle cell lymphoma; BL: Burkitt-like; FL: follicular lymphoma; CLL: chronic lymphocytic leukemia.

### Treatment and outcome

Treatment of LD/t-AML consisted in intensive chemotherapy (*n* = 48), azacitidine (*n* = 12), low dose cytarabine (*n* = 4), 6-mercaptopurine and low dose methotrexate (*n* = 3), or best supportive care (*n* =12). Only three patients underwent allogeneic stem cell transplantation in complete remission (CR) after intensive chemotherapy. Characteristics of patients treated with intensive chemotherapy or azacitidine are summarized in Table 4. In the group of patients treated by azacitidine, one patient achieved CR and another one achieved partial remission. Median OS from LD/t-AML and from LD diagnosis after azacitidine was 12.6 months and 157 months, respectively.

**Table 4:** Characteristics of LD/t-AML according to treatment

	Intensive chemotherapy *n* = 48	Azacitidine *n* = 12
Delay to secondary AML* – months (median, IQR)	60 (32–103)	123 (37–189)
**Center – n (%)**		
Toulouse	18 (37)	10 (83)
Marseille	30 (63)	2 (17)
**MDS prior to AML diagnosis – n (%)**	5 (10)	4 (33)
**Age at AML diagnosis - years (median, IQR)**	60 (51–67)	77 (70–78)
**ECOG PS – n (%)**		
0–1	25 (52)	10 (83)
2–3–4	9 (19)	0
Not known	14 (29)	2 (17)
**WBC - .10^9^/L (median, IQR)**	3.2 (1.8–33)	2.2 (1.4–7.5)
**Karyotype – n (%)**		
Favorable	3 (6)	0
Intermediate	24 (50)	7 (58)
Adverse	17 (35)	4 (33)
Unknown	4 (8)	1 (8)
**LD type – n (%)**		
DLBCL	9 (19)	2 (17)
MCL	2 (4)	0
BL	2 (4)	0
FL	13 (27)	2 (17)
CLL	6 (13)	1 (8)
MALT/SALT	1 (2)	1 (8)
Lymphocytic	0	0
Hodgkin lymphoma	8 (17)	2 (17)
T-cell lymphoma	2 (4)	1 (8)
**Stage at diagnosis – n (%)**		
Localized**/ Binet A	30 (63)	9 (75)
Advanced***/Binet B-C	10 (21)	1 (8)
Unknown	8 (17)	2 (17)
**Treatment – n (%)**		
Rituximab-containing regimen	18/43 (42)	5/10 (50)
Fludarabine-based	4/43 (9)	2/10 (20)
CHOP-like	20/43 (47)	4/10 (40)
Chlorambucil	10/43 (23)	2/10 (20)
Unknown	5 (8)	2 (17)
**No. of treatment lines (median, min-max)**	1 (1–5)	1 (1–3)
2 lines	12 (25)	1 (8)
≥ 3 lines	7 (15)	2 (17)
**Autologous stem cell transplantation – n (%)**	16 (33)	0

Abbreviations: MDS: myelodysplastic syndrome; NHL: non-Hodgkin lymphoma; WBC; white blood cell count.

*Defined as (AML diagnosis date – LD diagnosis date); **Localized is defined by Ann-Arbor classification 1–2; *** Advanced is defined by Ann-Arbor stage III-IV.

When considering patients treated with intensive chemotherapy, CR, early death, and treatment failure rates were 54%, 13%, and 33%, respectively. Among the 24 patients with intermediate-risk cytogenetics, 13 (54%) achieved CR as did 8 out of 17 patients (47%) with adverse cytogenetics. Median OS from LD/t-AML and from LD diagnosis were 7.7 and 75.6 months, respectively.

OS from t-AML diagnosis was highly variable according to the type of LD, with AML following treatment of DLBCL, HL and TCL displaying longer OS (73.0, 24.0 and 17.7 months, respectively), compared to OS of AML following treatment of MALT/SALT, CLL, MCL, BL and FL (1.8, 3.7, 4.3, 5.8 and 8.5 months, respectively – *P* = 0.02). Also, LD with advanced stages displayed shorter OS compared to localized stages (8.2 vs. 16.8 months, *P* = 0.23). Similarly, number of prior treatment lines for LD was associated with a shorter median OS (13.5, 7.9, and 6.0 months after 1, 2, or ≥ 3 treatment lines, respectively), although this difference was not statistically significant. In univariate analysis, age greater than 60 years (*P*= 0.002), ECOG performance status greater than 1 (*P* = 0.0002), myelodysplastic syndrome prior to LD/t-AML (*P* = 0.09), rituximab-containing regimens (*P* = 0.07), and fludarabine-based regimens or chlorambucil (*P* = 0.11) had a negative impact on OS (Table 5). In multivariate analyses for OS, only age (*P* = 0.04; HR 1.06 CI_95_[1.003–1.12]) and ECOG performance status 2–3 (*P* = 0.04; HR 8.0 CI_95_[1.25–51.6]) were independent prognostic factors in patients treated by intensive chemotherapy.

**Table 5:** Univariate analysis of overall survival for patients treated with intensive chemotherapy

Variable	Median OS^§^(months)	*p* (log-rank)
**Age**		0.002
< 60 years	16.4	
≥ 60 years	6.0	
**ECOG**		0.0002
0–1	16.4	
2–3	2.4	
Not known	10.1	
**Number of treatment lines**		0.74
1	13.5	
2	7.9	
≥ 3	6.0	
Karyotype*		0.22
Favorable	NR	
Intermediate	7.1	
Adverse	8.5	
Cytopenia prior to AML diagnosis**		0.25
Yes	16.4	
No	3.1	
MDS prior to AML diagnosis***		0.09
Yes	6.3	
No	9.4	
**White blood cell count at AML diagnosis**		0.30
< 10.10^9^/L	15.2	
≥ 10.10^9^/L	7.4	
**Stage at LD diagnosis**		0.23
Indolent / Binet A	16.8	
Aggressive / Binet B-C	8.2	
**Histological type of LD**		0.02
DLBCL	73.0	
MCL	4.3	
BL	5.8	
FL	8.5	
CLL	3.7	
MALT/SALT	1.8	
Hodgkin lymphoma	24.0	
T-cell lymphoma	17.7	
**Center**		0.24
Toulouse	7.3	
Marseille	8.5	
**Rituximab-containing regimen**		0.07
Yes	4.6	
No	15.2	
**Chemotherapy type**		0.11
CHOP-like	15.0	
Fludarabine-based	5.3	
Chlorambucil	7.9	
**Autologous stem cell transplantation**		0.88
Yes	7.9	
No	9.3	

Abbreviations: NR: not reached; MDS: myelodysplastic syndrome; NHL: non-Hodgkin lymphoma.

* According to MRC classification [37].

** Defined as anemia, thrombocytopenia, neutropenia or any combination after end of LD treatment and more than six months before AML diagnosis – lymphopenia excluded.

*** Defined as documented MDS diagnosed more than six months before AML diagnosis.

§ from AML diagnosis.

### Outcome of LD/t-AML compared to other subtypes of secondary and t-AML

**Figure 1:**
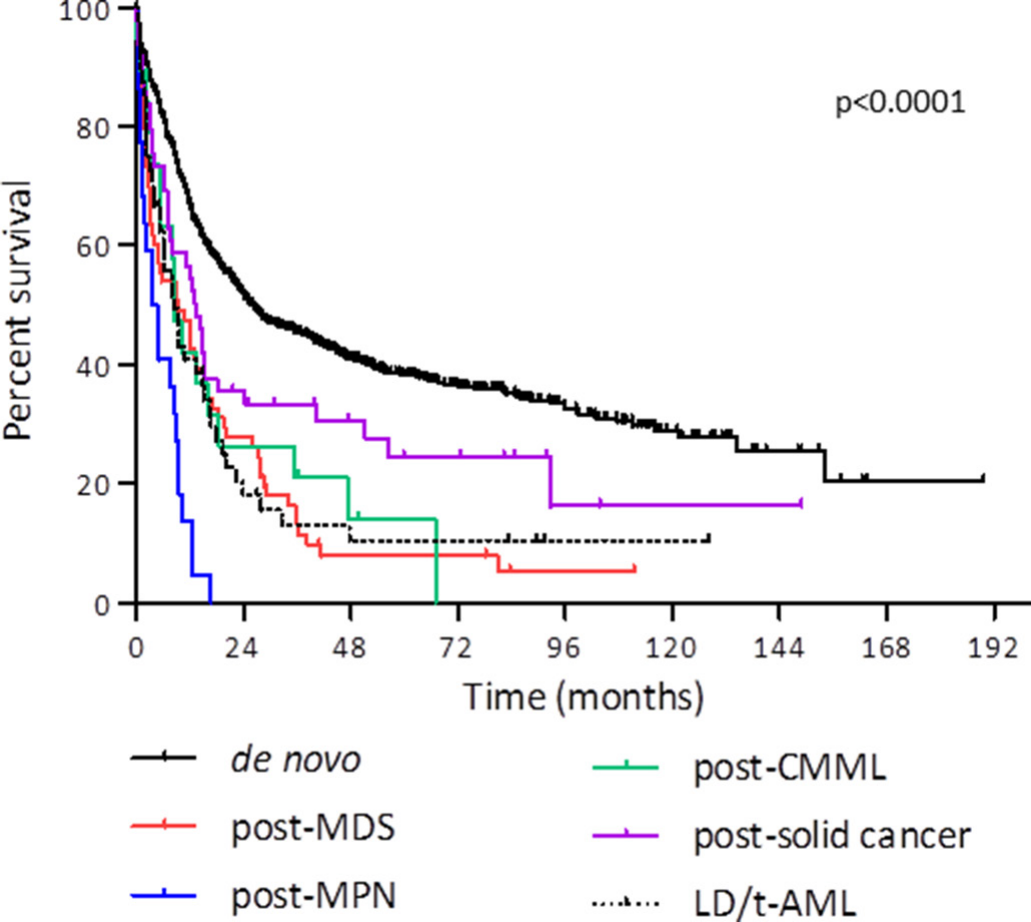
Overall survival after intensive chemotherapy according to AML type

Patients of the Toulouse University Hospital database treated by intensive chemotherapy in the same study period (*n* = 853) had a median OS of 19.7 months. Median OS was 26.1 months in *de novo* AML (*n* = 663), 4.2 months in post-Philadelphia negative myeloproliferative neoplasm AML (*n* = 22), 9.4 months in post-myelodysplastic syndrome AML (*n* = 64), 8.6 months in post-chronic myelomonocytic leukemia AML (*n* = 19), 13.4 months in t-AML following the treatment of solid cancer (*n* = 50), and 14.7 months when focusing on breast cancer only (*n* = 30) (Figure 1).

## DISCUSSION

This study shows that t-AML following the treatment of lymphoid malignancies is heterogeneous in terms of delay of onset, cytogenetic risk, response to therapy, and outcome. The recent introduction of rituximab in B-cell malignancies did not appear to change the characteristics of LD/t-AML although its delay of onset was shorter in patients who received this monoclonal antibody. Overall, prognosis of patients treated by intensive chemotherapy remains very poor.

t-AML accounted for 10.6% of all cases of AML treated with intensive chemotherapy in this period in Toulouse University Hospital, and LD/t-AML accounted for 4.1% of all cases and for 18.4% of secondary AML (including post-MDS and post-MPN AML). This result is slightly higher than the incidence reported by the Danish and Swedish registries and by the German-Austrian AMLSG group [11, 13, 18].

Despite obvious pitfalls including heterogeneity of LD subtypes and treatments, a low number of patients, and missing data due to the long period of inclusion, this retrospective study describes all consecutive cases of AML secondary to LD treatments in a recent period and contributes to define clinical characteristics and prognostic factors for this particular subtype of t-AML since most studies included various previous cancer including but not limited to LD. As expected, we found an overrepresentation of *MLL* rearrangements, complex and monosomal karyotypes, compared to *de novo* AML. In particular, FAB-M6 AML was found in 11% of cases, which is unusual in large series of AML and significantly higher compared to *de novo* AML (3%).

We have identified distinct profiles of LD/t-AML according to the LD subtype. Indeed, longer latency and better OS were observed in HL patients, whereas TCL or aggressive NHL patients had a shorter time to t-AML and CLL patients had the lowest median overall survival. Previous treatments and subsequent genomic or microenvironment alterations, altered immune background related to the underlying LD and treatment, age at LD diagnosis (which was a major determinant for delay from LD diagnosis to LD/t-AML diagnosis as already reported) [19], or genetic susceptibilities including polymorphisms in genes governing drug metabolism, DNA repair, and leukemogenesis, may account for these distinct features [5, 20]. T-AML are genetically characterized by abnormal double-strand break repair and frequent *TP53* mutations selected by previous chemotherapy, predisposing to genomic instability [9, 21–22]. Indeed, the large majority of t-AML shows cytogenetic abnormalities indicating that chromosomal aberrations may constitute the main driver of disease in these patients [23]. In our series, few cases were molecularly annotated with *FLT3*-ITD found in only two of 17 cases (11.8%) and *NPM1* mutation in one of 15 (6.7%). A recent study has established a genetic classification of t-AML through the sequencing of 82 genes suspected to be involved in the pathogenesis of myeloid malignancies [24]. Unfortunately, we did not provide data on *SRSF2, SF3B1, U2AF1, ZRSR2, ASXL1, EZH2, BCOR, STAG2* or *TP53* mutations since the sequencing of these genes was not performed throughout the study period. Additional studies are needed to determine the distribution of theses mutations and provide new elements for better understanding the pathogenesis of t-AML following lymphoid malignancies.

The risk of AML following B-CLL treatment remains controversial since some studies did not report such an association [16]. It has been suggested that the association of fludarabine with an alkylating agent may increase the incidence of t-AML and perhaps to a greater extent when combined with rituximab [25–27]. After frontline fludarabine-cyclophosphamide-rituximab (FCR) treatment for B-CLL, latency of 35 months to develop t-AML or MDS was reported. These therapy-related myeloid neoplasms could emerge directly from prolonged myelosuppression following FCR or after achieving complete hematological recovery with latency significantly shorter in the former group (23 months vs. 42 months) [27]. However, these studies should be interpreted cautiously since the CLL-8 randomized study comparing FCR to FC did not show any difference in the incidence of secondary AML after a median follow-up of 5.9 years [28].

Intensive chemotherapy in patients with AML secondary to previous MDS, CMML, MPN, or prior cytotoxic exposure remains unsatisfactory compared to *de novo* AML [13, 19]. However, our results suggest that t-AML following treatment of solid cancer has a better outcome compared to post-MDS, post-CMML, and post-LD while post-MPN AML seems to have the worst outcome. Azacitidine has emerged as an alternative of intensive chemotherapy, especially in poor-risk patients [29–30]. Azacitidine has already been evaluated in therapy-related myeloid neoplasms with 40% overall response rate and a median OS between 9 and 21 months [31–33], which is in line with our LD/t-AML series, although the number of patients treated in our study was very low.

In conclusion, the prognosis of LD/t-AML is particularly poor whatever the treatment. Prevention of AML emergence by using a less leukemogenic regimen for lymphoid malignancies should be carefully weighed at LD diagnosis. New treatment strategies in patients with LD/t-AML and other secondary AML are urgently needed.

## MATERIALS AND METHODS

### Patient selection

Our study included 80 consecutive cases of t-AML secondary to NHL, HL, T-cell lymphoma and chronic lymphocytic leukemia diagnosed and treated in Toulouse University Hospital and Institut Paoli-Calmettes (Marseille) between 1997 and 2012. All patients were treated for their LD. AML secondary to myeloma or acute lymphoblastic leukemia were not considered in this study. Written informed consent was obtained from all patients in accordance with the Declaration of Helsinki, allowing the collection of clinical and biological data in an anonymized database. The cytogenetic classification for AML was in accordance with the Medical Research Council classification [33]. Treatment of AML has been described previously [30, 35–37].

### Statistical analyses

We described patient characteristics using number and frequency for qualitative data and median, and interquartile range (IQR) for quantitative data. Differences were tested with Chi2 test (or Fisher’s exact test in case of small expected numbers) for qualitative data and with Student *t*-test (or Mann-Whitney U test) for quantitative data. Complete response referred to the combination of complete response (CR) and complete response with incomplete blood count recovery (CRi) defined by international consensus criteria [38]. Early death was defined as death from any cause occurring between the start of chemotherapy and the response assessment. Treatment failure was defined as failure to achieve CR with evidence of persistent leukemia by blood and/or bone marrow examination. Overall survival (OS) was measured from the date of diagnosis to the date of death from any cause; patients not known to have died at last follow-up were censored on the date they were last known to be alive. Survival functions were estimated by the Kaplan-Meier method and differences were tested using the log-rank test. Multivariate analysis of OS was conducted using the Cox model. Age, ECOG performance status, white blood cell count (WBC) and karyotype at diagnosis, myelodysplastic syndrome prior to AML diagnosis, stage at LD diagnosis, histological type of LD, type of treatment, number of previous treatment lines, autologous stem cell transplantation, and center of diagnosis were included in the model. All reported *P* values were two-sided, and the significance threshold was < 0.05. Statistical analyses were performed on GraphPad Prism and STATA v13.
